# Walking on the Moon: A randomized clinical trial on the role of lower body positive pressure treadmill training in post-stroke gait impairment

**DOI:** 10.1016/j.jare.2019.09.005

**Published:** 2019-09-19

**Authors:** Rocco Salvatore Calabrò, Luana Billeri, Veronica Agata Andronaco, Maria Accorinti, Demetrio Milardi, Antonino Cannavò, Enrico Aliberti, Angela Militi, Placido Bramanti, Antonino Naro

**Affiliations:** a Robotic Neurorehabilitation Unit, IRCCS Centro Neurolesi Bonino Pulejo, Messina, Italy; b Department of Biomorphology and Biotechnologies, University of Messina, Messina, Italy; c Department of Motor Sciences, University of Messina, Messina, Italy

**Keywords:** AlterG, Lower body positive pressure support system, Gait training, Conventional treadmill gait training, Stroke

## Abstract

Body weight–supported treadmill training (BWSTT) can be usefully employed to facilitate gait recovery in patients with neurological injuries. Specifically, lower body positive pressure support system (LBPPSS) decreases weight-bearing and ground reaction forces with potentially positive effects on qualitative gait indices. However, which gait features are being shaped by LBPPSS in post-stroke patients is yet poorly predictable. A pilot study on the effects of LBPPSS on qualitative and quantitative gait indices was carried out in patients with hemiparesis due to stroke in the chronic phase. Fifty patients, who suffered from a first, single, ischemic, supra-tentorial stroke that occurred at least 6 months before study inclusion, were enrolled in the study. They were provided with 24 daily sessions of gait training using either the AlterG device or conventional treadmill gait training (TGT). These patients were compared with 25 age-matched healthy controls (HC), who were provided with the same amount of AlterG. Qualitative and quantitative gait features, including Functional Ambulation Categories, gait cycle features, and muscle activation patterns were analyzed before and after the training. It was found that AlterG provided the patients with higher quantitative but not qualitative gait features, as compared to TGT. In particular, AlterG specifically shaped muscle activation phases and gait cycle features in patients, whereas it increased only overall muscle activation in HC. These data suggest that treadmill gait training equipped with LBPPSS specifically targets the gait features that are abnormal in chronic post-stroke patients. It is hypothesizable that the specificity of AlterG effects may depend on a selective reshape of gait rhythmogenesis elaborated by the locomotor spinal circuits receiving a deteriorated corticospinal drive. Even though further studies are warranted to clarify the role of treadmills equipped with LBPPSS in gait training of chronic post-stroke patients, the knowledge of the exact gait pattern during weight-relief is potentially useful to plan patient-tailored locomotor training.

## Highlights

• The effects of LBPP on locomotion in neurologic patients are poorly predictable.
• The mechanisms through which LPBB acts on gait are partially unknown.
• Gait training using AlterG improves functional gait in post-stroke patients.
• AlterG increases muscle activation and/or phasic muscle activation in post-stroke.
• This knowledge may be useful to plan patient-tailored LBPP locomotor training.

## Graphical abstract

**Figure:**
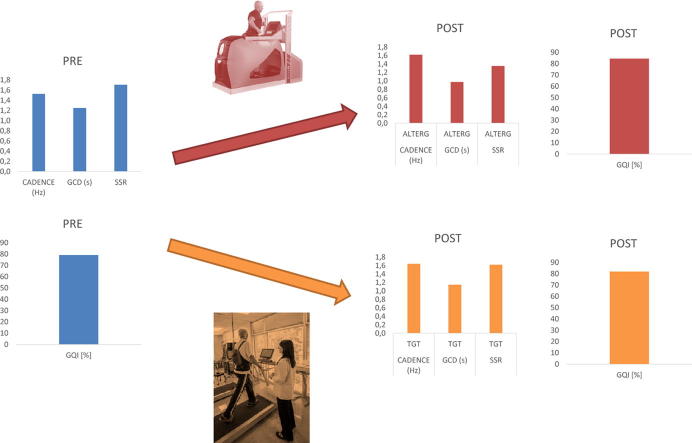

## Introduction

Employing treadmill training in gait rehabilitation can be of significant help in achieving functional ambulation in patients with neurological damage, including stroke. In fact, treadmill training augments the ability to walk independently of patients with stroke, although in the short term, and provide them with higher walking speed and walking endurance as compared to traditional overground gait training. These effects are magnified further when coupling treadmill training with BWS (body weight–supported treadmill training - BWSTT) [1], [2], [3], [4], [5], [6]. Indeed, BWSTT can alleviate post-stroke survivors’ weight bearing and effort (and physiotherapist’s effort) during gait training, allowing the patient to walk when muscle strength and postural control are still non-sufficient for functional ambulation. Consequently, they may allow for mobilization and training early [7], [8]. Last, BWSTT is useful because it provides for walking with reduced ground reaction forces and normal ranges of motion of lower limb joints [9]. Altogether, these aspects of BWSTT may facilitate the improvement in qualitative and quantitative gait indices, although controversial data is available [6], [10], [11], [12], [13], [14], [15], [16], [17], [18], [19]. However, some innovative BWSTTs give the patient a from-below lifting force by employing lower body positive pressure support system (LBPPSS). These systems implement differential air pressure technology using a chamber to reduce the weight of an individual while walking up to 100% of the original body weight, instead of using a body-suspension harness system.

LBPPSS is increasingly used after knee surgery to reduce ground reaction forces during walking and running to facilitate postoperative rehabilitation [20], [21]. It has also been successfully employed in children with cerebral palsy [22]. Conversely, there are no data on LBPPSS usefulness in post-stroke rehabilitation. There are several issues to be although considered before employing LBPPSS in post-stroke patients. The use of LBPPSS may be relatively contraindicated in such patients, given that LBPP can affect systemic blood pressure and cerebral blood flow [23]. Further, whether LBPPSS can affect kinematic (including spatiotemporal variables) and kinetic parameters of gait still has to be clearly determined [23], [24], [25], [26], [27], [28], [29], [30], [31], [32], [33], [34]. In fact, LBPP may generate unwanted horizontal assistance due to the interface between the chamber and the subject, thus irregularly modifying locomotion kinematics and kinetics [25]. In addition, there is no clear correlation between BWS and the results of ground-reaction forces during overground walking with different percentages of BWS [29], [30]. Moreover, LBPP would influence only the stance phase. In fact, the swinging limb remains subject to full gravity given that it cannot be pulled in proportion to the simulated gravity level [35]. Therefore, muscle activity decreases as BWS increases without, however, any proportionality [35]. Finally, the metabolic cost of BWS has still to be clearly determined [36], [37].

To synthesize, the effects of LBPPSS on gait kinematic variables could be neither predictable nor necessarily useful to recovering functional gait in post-stroke patients. Indeed, whether improvements in temporal variables of gait correspond to progress in functional gait is unknown. This study was aimed at offering a preliminary estimation of the safety and the effectiveness of the LBPPS AlterG (AlterG Inc.; Fremont, CA, USA) on temporal variables of gait and on functional ambulation measures in a sample of patients with hemiparesis due to stroke in the chronic phase. The hypothesis was that LBPPSS would significantly improve functional gait in comparison to conventional treadmill gait training (TGT) thanks to specific, gait phase-related, changes in temporal variables of gait and muscle activations.

## Materials and methods

### Experimental procedure

The study was designed as a single-blind, prospective, randomized controlled trial (RCT) to compare the effects of LBPPSS (provided using AlterG) and TGT in patients with stroke. Clinical and gait data of the patients were compared with those of 25 age-matched (by a frequency-matching approach) healthy controls (HC).

### Participants

Fifty patients among the 250 attending the Robotic Neurorehabilitation Unit of the institute in 2018 were enrolled in the study according to the following inclusion criteria: (i) age ≥ 55 years; (ii) first, single, ischemic supra-tentorial stroke occurred at least 6 months before study inclusion; (iii) a Functional Ambulatory Categories (FAC) score of ≥ 2; (iv) the ability to control head and trunk posture; (v) no systemic or cardiovascular contraindication to LBPP; and (vi) the ability to give personal consent, understand instructions and learn through practice (Abbreviated Mental Test > 7/10). The exclusion criteria were as follows: (i) recurrent stroke; (ii) recent brain surgery; (iii) spasticity of modified Ashworth scale greater than 3; (iv) fixed contracture of any lower limb joint or painful joints; (v) ataxia, dystonia, or tremor of lower limbs; (vi) cervical myelopathy; (vi) severe aphasia; and (vii) a history of seizures in the past 12 months. The study also included a sample of 25 HC (i.e., without any evidence of neurological, psychiatric, cardiovascular, orthopedic, or systemic disease). The institutional review board approved the study (IRCCSME#19/17); all participants gave their written informed consent to study inclusion.

### Intervention

Patients were randomized into two groups (with a 1:1 allocation ratio). A group practiced one session a day of AlterG (for 40 min), six days a week, for four weeks (for a total amount of 24 sessions). The other group practiced one session a day of TGT (for 40 min), six days a week, for four weeks (for a total amount of 24 sessions). HCs were provided with one session a day of AlterG (for 40 min), six days a week, for four weeks (for a total amount of 24 sessions).

The LBPPSS AlterG consists of a treadmill with handrails equipped with a waist-high inflatable chamber. The subject wears neoprene shorts that zip into the chamber, creating an airtight seal around the subject's waist. During training, positive pressure inflates the chamber, and the difference in pressure around the waist seal produces a lifting force [26]. The LBPP makes the patient feel more comfortable than overhead harness systems to support body weight, and it allows for a kinematic walking pattern similar to overground walking. The subject can walk freely or use the handrails of the treadmill, with physiotherapist supervision.

The patients undergoing AlterG were trained with the assistance/supervision of a trained physiotherapist depending on the patient’s FAC score (FAC 2: to walk with the intermittent support of one physiotherapist to help with balance and coordination; FAC 3: to walk with the visual supervision of one physiotherapist; FAC 4: to walk independently without using the handrails). BWS, physiotherapist assistance, and treadmill speed (TS) were checked and adapted to subjects’ progress in terms of FAC scoring across the AlterG sessions. Also, the participants who practiced TGT were trained using a FAC-tailored approach. The HC initially practiced the AlterG at the same amount of BWS and TS administered to the patients. BWS and TS were reduced and increased, respectively, in pre-established steps across the AlterG sessions. It was necessary to provide also HC with LBPP to have a better reference value, given that even healthy people can display normal variations from the normal pattern of walking.

### Outcomes

All outcome measures were obtained the day before and the day after the training, so to avoid any interference on the training and biasing effect of fatigue. The primary outcome was the FAC score for the qualitative gait assessment. FAC is a 6-point scale (rating from 0 to 5) that evaluates ambulation status by determining how much human support the patient requires when walking, regardless of assistive device use. A score of zero indicates that the patient cannot walk (non-functional ambulation); a score of one denotes a dependent ambulator who requires assistance from another person in the form of continuous manual contact; a score of two indicates continuous or intermittent manual contact; a score of 3 verbal indicates supervision/guarding. Scores of four and five describe patients who can walk freely only on level surfaces or on any surface, respectively (independent ambulation).

The secondary outcomes were the temporal parameters of gait and the dynamic electromyography data. Specifically, the gait cycle features and muscle activation were quantified while the participant walked overground using an eight-channel wireless system (FreeEMG1000 system; BTS Bioengineering, Milan, Italy) equipped with an accelerometer (G-Sensor). As outcome measures, the step time (i.e., the time between the heel strike of one leg and the heel strike of the contralateral leg), the stance/swing ratio (SSR, that is, the ratio between swing time -the time during which the foot is not in contact with the floor- and stance time -the time during which the foot is in contact with the floor), the cadence (i.e., the number of steps per second), and the Gait Quality Index (GQI, which estimates the overall deviation from the average gait of a control population by using the temporal parameters) were quantified [38], [39].

The duration of the gait cycle was normalized to 100% to calculate the root-mean-square (RMS) amplitude of each muscle (a temporal parameter estimating muscle activation), so to make the comparisons among conditions and subjects possible. Thus, the mean RMS was computed by averaging 10 RMS values related to 10% partitions of the gait cycle across the subjects. We also computed the overall RMS over the entire walking trial without partitioning. All of these measurements were corrected for the Froude number (*Fr*) to normalize for differences in dynamic behavior. *Fr* is calculated as the ratio of the square of the TS to the length of the lower limb (L) from the greater trochanter to the ground, and the acceleration due to gravity (g), according to the formula TS^2^/(g × L) [40].

Surface myoelectric signals were sampled at 1000 Hz from rectus femoris (RF), biceps femoris (BF), tibialis anterior (TA), and gastrocnemius medialis (G) of both lower limbs. After careful preparation of the skin, the bipolar adhesive surface electrodes were placed over the muscle belly in the direction of the muscle fibers according to the European recommendations for surface electromyography (SENIAM). This was done to ensure repeatable electrode placement during the training [41], [42], [43]. Meanwhile, the EMG signals were processed to obtaining RMS values using Smart Analyzer software v.1.10.469.0 (BTS Bioengineering; Milan, Italy), so to investigate lower limb muscle activation as modified by training interventions [44].

### Sample size, randomization, blinding

Twenty patients per arm would have been required to observe a minimum median improvement (±IQR) of +1(1) scale-point for the FAC [45], with α = 0.05 and 1 − β = 0.8. Twenty-five patients were thus recruited per arm to allow for dropouts.

The randomization procedures were carried out thanks to a computer-generated list covered by straps to conceal the allocation.

The experimenters who assessed the patients and analyzed the data were blind on patients’ allocation.

### Statistical methods

All data were described quantitatively using median (with IQR) and mean (with standard deviation) where appropriate. Clinical data changes over time were assessed using the Wilcoxon test. A Bonferroni adjustment for the two time points was made (α = 0.025). Between-group comparison was carried out using the Mann-Whitney test.

The secondary outcomes were assessed using a multivariate analysis of covariance (MANCOVA) to reduce the probability of type I error owing to multiple comparisons [46]. *Post-hoc* analysis with univariate 3-way ANCOVA with the factors *time* (two levels: before and after the training), *lower-limb* (three levels for *group × lower-limb × time* data analysis: affected, unaffected, and healthy limbs; datasets related to healthy limbs were pooled together; and two levels for *lower-limb × time* data analysis: affected vs. unaffected in the patients, or left vs. right in HC), and *group* (three levels: AlterG, TGT, and HC) was used to indicate which temporal measure showed significant changes.

Concerning RMS, the average EMG data from each 10% partition of the entire gait cycle in each muscle and the overall RMS of the entire gait cycle in each muscle were analyzed using a univariate 2-way ANCOVA with the factors *time* (two levels: before and after the training) and *group* (three levels: AlterG, TGT, and HC).

Clinical and demographic characteristics (age, gender, affected side, and disease duration) and comorbidities (Table 1) were added to the analysis as covariates. The *effect size* (E) of each outcome measure was defined as small (<0.41), medium (0.41 to 0.70), or large (>0.70) to estimate the effect of the AlterG treatment. An α-level of *P* < 0.05 was assumed to be significant, and the Bonferroni correction was then used for *post-hoc* comparisons. With regard to the factor *lower-limb*, datasets related to healthy limbs and affected/unaffected limbs were pooled in separate sessions to compare the affected and unaffected sides of patients and to compare the affected and unaffected side of patients to the healthy limbs [47], [48]. Multiple linear regression was used to determine the strength of correlation between TS, BWS, and peak muscle activation.

**Table 1:** Clinical-demographic characteristics of patients provided with AlterG, treadmill gait training (TGT), and of healthy controls (HC).

Parameters		AlterG (n = 25)	TGT (n = 25)	HC (n = 25)
age in years, mean (sd)		65 (6)	62 (5)	62 (6)
gender (female/male)		15/10	17/10	12/13
paretic limb (right/left)		17/8	14/11	
months from stroke, mean (sd)		9 (2)	8 (4)	
FAC, median (IQR)		3 (2–4)	3 (2–4)	
comorbidities (n)	none	4	3	
	dyslipidemia	12	10	
	diabetes mellitus	6	8	
	alcoholism/smoking	3	2	
	blood hypertension	15	18	

## Results

At baseline, all patients required a degree of assistance from the physiotherapist while walking corresponding to an FAC of 3 (IRQ 2–4). Patients showed a lower cadence in comparison to HC (*P* < 0.001). Moreover, a longer step time with the affected side and a shorter step time with the unaffected limb were appreciable (lower limb comparison, *P* < 0.001; each patient’s lower limb in comparison to HC, *P* < 0.001). This was paralleled by a lower SSR in the affected lower limb and a higher SSR in the unaffected limb (lower limb comparison, P < 0.001; each patient’s lower limb in comparison to HC, *P* = 0.01). The mildness of SSR changes in comparison to HC depended on the fact that the percent gait cycle duration was longer in the patients with stroke compared to HC. Last, patients showed a lower GQI in both lower limbs (lower limb comparison, *P* < 0.001; each patient’s lower limb in comparison to HC, *P* < 0.001) (Fig. 2). There were no significant differences between TGT and AlterG groups, as well as no pair-wise lower limbs differences were appreciable between the patient groups. HC showed ho significant inter-limb differences. Both groups had a lower activity of the affected TA and BF, a higher activity of the affected G in the 20, 30, 40, and 50% of the gait cycle, and a higher activity of the affected RF in the 50, 60, and 70% of the gait cycle, as compared to HC (each comparison *P* < 0.001) (Fig. 3). On average, the patients who practiced AlterG required a BWS of 65 ± 10% and a TS of 0.53 ± 0.1 m/s at the beginning of the training (Fig. 2). HCs were provided with the same amount of BWS and TS.

**Fig. 1:**
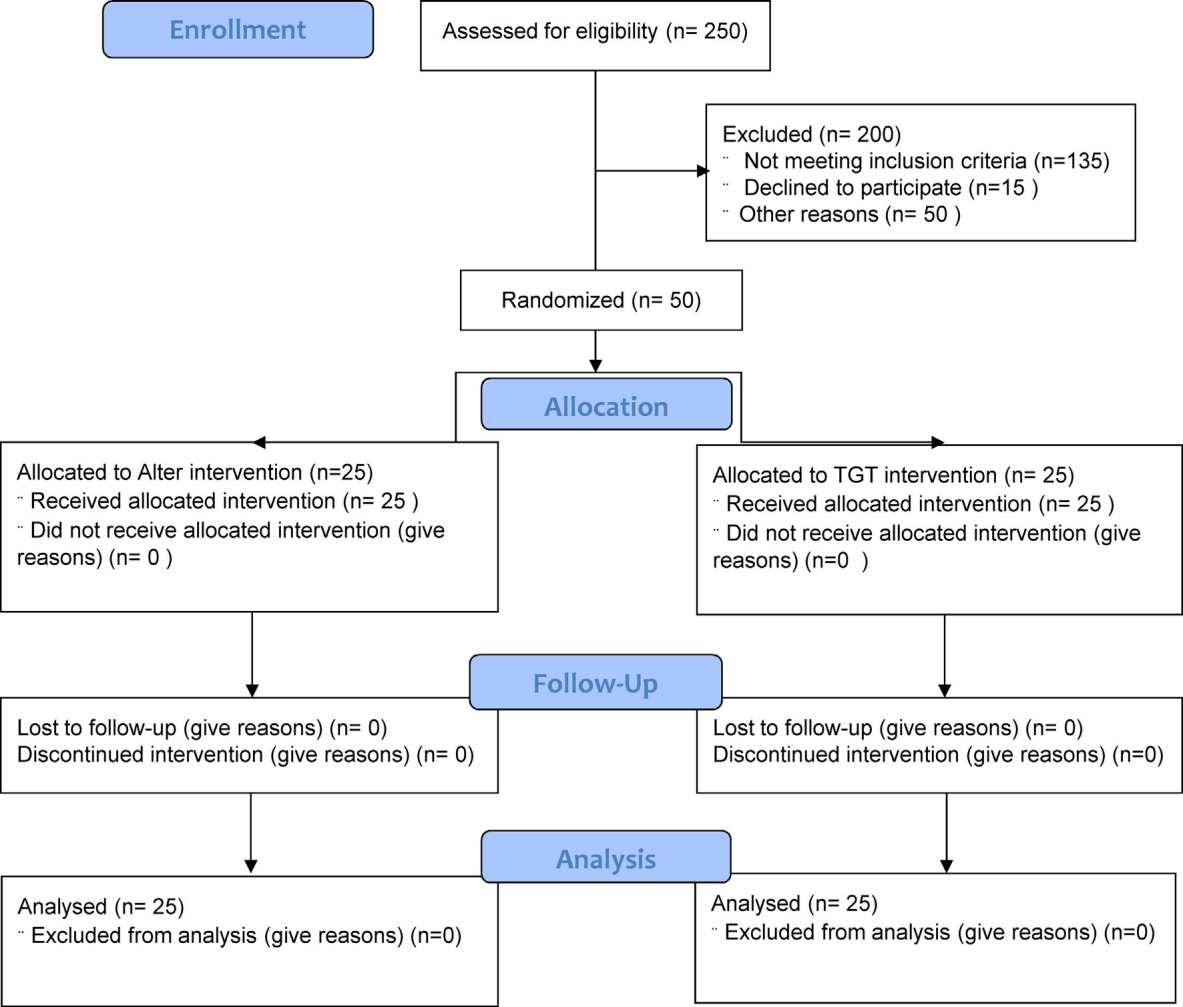
Flow diagram.

**Fig. 2:**
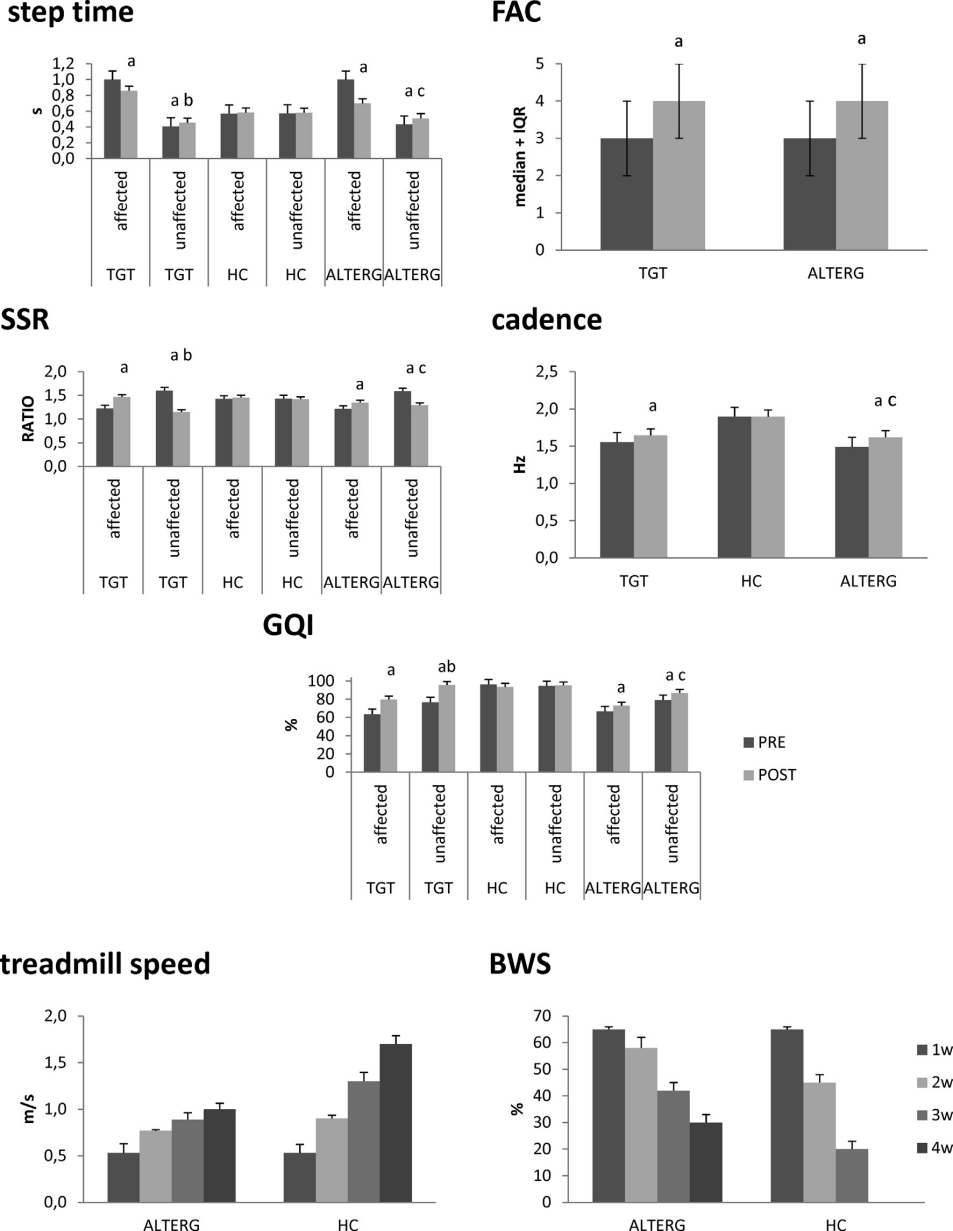
Mean values of Functional Ambulation Category (FAC), body weight support (BWS), speed of treadmill, cadence, step time, stance-swing ratio (SSR), and gait quality index (GQI) for each group (AlterG, treadmill gait training –TGT, and healthy controls –HC). Within-group post-pre changes are indicated by letter a, inter-limb difference by letter b, and between-group changes by letter c. Vertical bars indicate standard deviation. Statistical data are detailed in table 2.

**Fig. 3:**
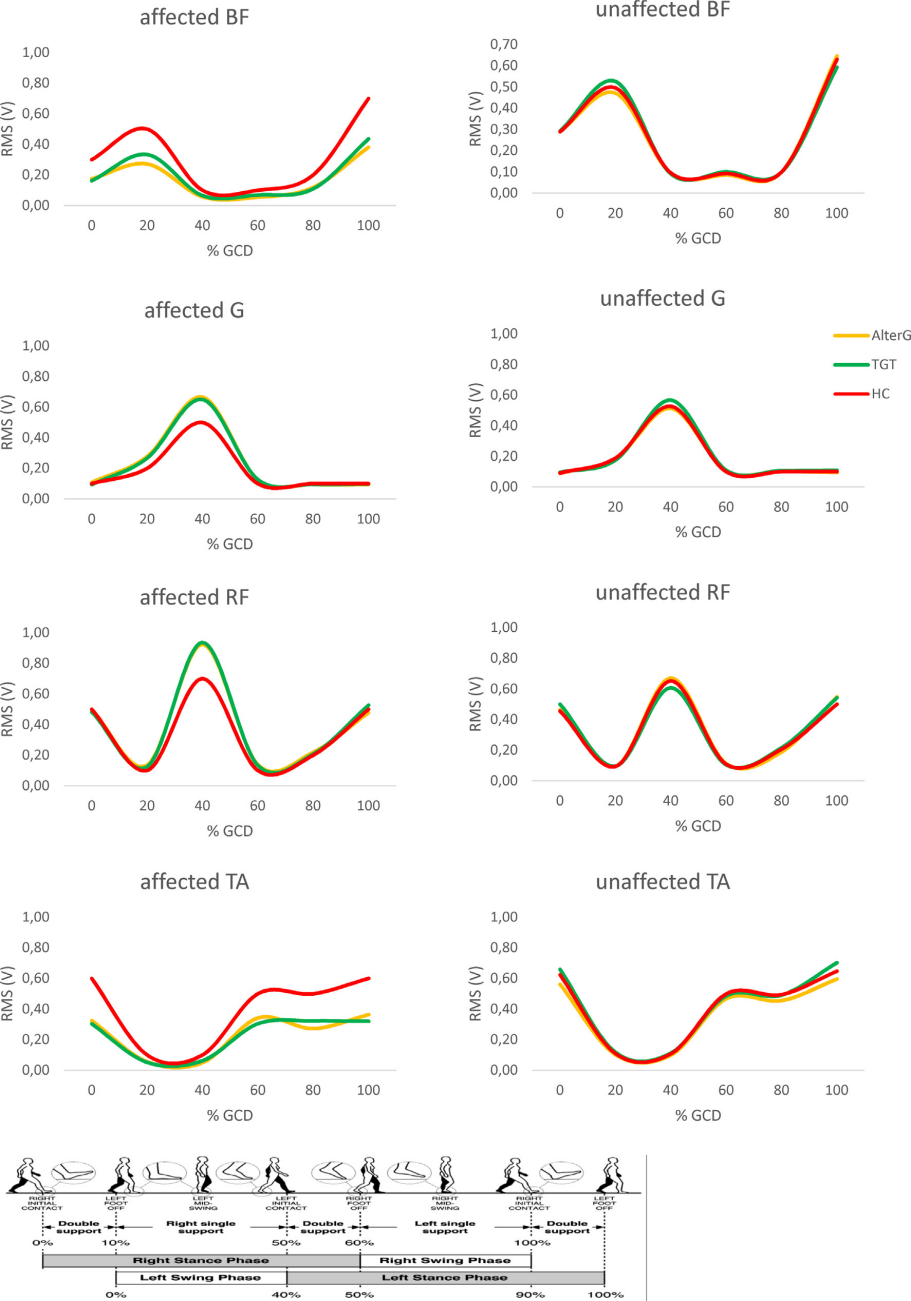
Mean EMG activity computed over the normalized gait cycle before gait training in patients (AlterG and treadmill gait training, TGT) and healthy controls (HC). RMS values (V) are shown for gastrocnemius, G, rectus femoris, RF, biceps femoris, BF, and tibialis anterior, TA, of affected and unaffected lower limb.

All enrolled participants completed the trial, without reporting any side effects or adverse events (Fig. 1).

BWS was progressively scaled down to 30 ± 10% in patients provided with AlterG, whereas FAC was adapted to the subject’s need during TGT. TS was progressively scaled up to 1 ± 0.2 m/s in patients undergoing AlterG, and 0.79 ± 0.1 m/s in patients undergoing TGT. Indeed, post-training FAC increased by at least one scale-point in both AlterG and TGT groups without significant between-group differences (Fig. 2, Table 2). BWS and TS in the HC group were instead progressively scaled down to 0% in daily steps of 3%, and scaled up to 1.73 m/s, in daily steps of 0.05 m/s (Fig. 2).

**Table 2:** Statistical data of training aftereffects on clinical scale and gait temporal parameters (see Fig. 2). Non-significant data are not reported. Concerning *post-hoc t-*tests, lower limbs of HCs were pooled together and compared with the affected and unaffected lower limb of patients.

*group × time* *P*-value [E]		*Time* *P*-value [E]				*Post-hoc t-*tests *P*-value [E]		
FAC	ns	AlterG	*P* < 0.001 [0.9]			AlterG vs. TGT	ns	
		TGT	*P* < 0.001 [0.9]					
cadence	*P* = 0.01 [0.8]	HC	ns			HC vs. AlterG	*P* < 0.001 [0.9]	
		AlterG	*P* = 0.005 [0.9]			HC vs. TGT	*P* < 0.001 [0.9]	
		TGT	*P* = 0.01 [0.8]			AlterG vs. TGT	*P* < 0.001 [0.9]	

*group × lower-limb × time* *P*-value [E]		*lower-limb × time* *P*-value [E]				*Post-hoc t-*tests *P*-value [E]		
GQI	*P* = 0.01 [0.8]	HC	ns	left	ns	HC vs. AlterG	affected	*P* < 0.001 [0.9]
				right	ns		unaffected	*P* < 0.001 [0.9]
		AlterG	ns	affected	*P* < 0.001 [0.9]	HC vs. TGT	affected	*P* < 0.001 [0.9]
				unaffected	*P* < 0.001 [0.9]		unaffected	*P* < 0.001 [0.9]
		TGT	*P* < 0.001 [0.9]	affected	*P* < 0.001 [0.9]	AlterG vs. TGT	affected	*P* < 0.001 [0.9]
				unaffected	*P* < 0.001 [0.9]		unaffected	*P* < 0.001 [0.9]
step time	*P* < 0.001 [0.9]	HC	ns	left	ns	HC vs. AlterG	affected	*P* < 0.001 [0.9]
				right	ns		unaffected	*P* < 0.001 [0.9]
		AlterG	ns	affected	*P* < 0.001 [0.9]	HC vs. TGT	affected	*P* < 0.001 [0.9]
				unaffected	*P* = 0.01 [0.7]		unaffected	*P* < 0.001 [0.9]
		TGT	*P* < 0.001 [0.9]	affected	*P* < 0.001 [0.9]	AlterG vs. TGT	affected	*P* < 0.001 [0.9]
				unaffected	*P* < 0.001 [0.9]		unaffected	*P* < 0.001 [0.9]
SSR	*P* < 0.001 [0.9]	HC	ns	left	ns	HC vs. AlterG	affected	*P* < 0.001 [0.9]
				right	ns		unaffected	*P* < 0.001 [0.9]
		AlterG	ns	affected	*P* < 0.001 [0.9]	HC vs. TGT	affected	*P* < 0.001 [0.9]
				unaffected	*P* < 0.001 [0.9]		unaffected	*P* < 0.001 [0.9]
		TGT	*P* < 0.001 [0.9]	affected	*P* < 0.001 [0.9]	AlterG vs. TGT	affected	*P* < 0.001 [0.9]
				unaffected	*P* < 0.001 [0.9]		unaffected	*P* < 0.001 [0.9]

Legend: treadmill gait training, TGT; healthy controls, HC; [E] effect size; FAC Functional Ambulatory Categories; GQI Gait Quality Index; SSR stance/swing ratio.

Cadence increased more evidently after the AlterG training compared to TGT (Fig. 2, Table 2). GQI increased more evidently and in both lower limbs after the AlterG training compared to TGT, which instead showed a significant inter-limb difference (Fig. 2, Table 2). Step time and SSR partially reverted the baseline trend. Specifically, these parameters varied more evidently and in both lower limbs after the AlterG training compared to TGT, which also yielded a significant inter-limb difference (Fig. 2, Table 2).

HC did not show any significant change in gait features following AlterG training (Fig. 2, Table 2). Each *lower-limb* and *group* comparison over time between the patient groups and the HCs was thus significant.

**Fig. 4:**
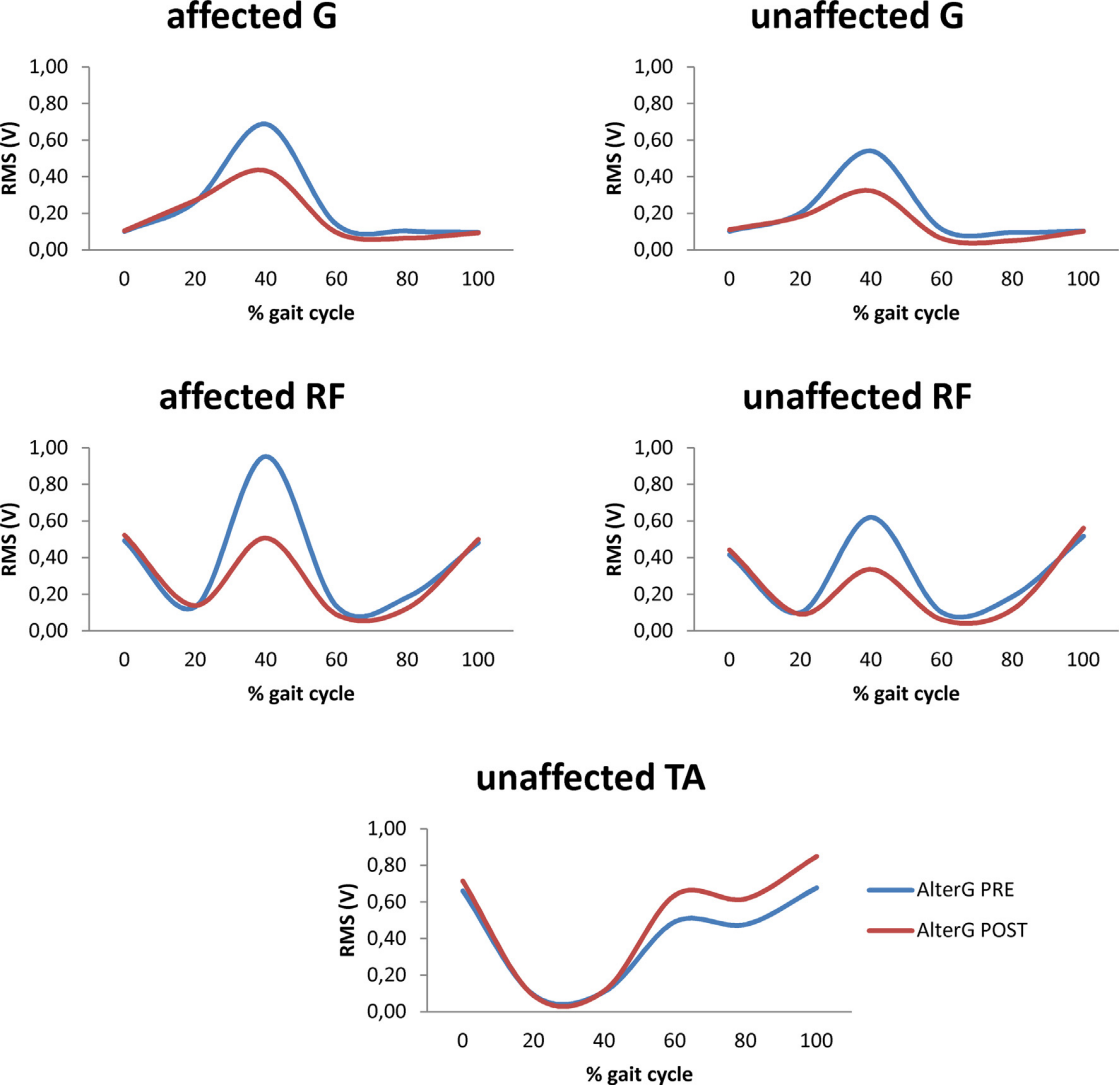
Mean EMG activity computed over the normalized gait cycle before (PRE) and after the end of AlterG gait training (POST) in patients (only significant changes are reported). RMS values (V) are shown for gastrocnemius, G, rectus femoris, RF, biceps femoris, BF, and tibialis anterior, TA, of affected and unaffected lower limb. Statistical data are reported in Table 3.

The treatments yielded significant effects on the target muscles. Specifically, AlterG in patients decreased the RMS in the 50, 60, and 70% of gait cycle in both G and both RF (Fig. 4), with comparable statistical data among these 10% partitions (Table 3). On the other hand, AlterG increased the RMS in the 70, 80, 90, and 100% of the gait cycle in the unaffected TA (Fig. 4), with comparable statistical data among these 10% partitions (Table 3). AlterG also increased the overall RMS more than AlterG in HC and TGT did; moreover, AlterG in HC and TGT yielded only an overall RMS increase. Statistical data are summarized in Table 3.

**Table 3:** Statistical data of training aftereffects on RMS (see Fig. 3). Non-significant data are not reported.

*group × time* p-value, [E]		*time* p-value, [E]		*post-hoc t-*tests p-value [E]	
aff G 50–70% GCD	P < 0.001 [0.9]	HC	ns	HC vs. AlterG	P = 0.003 [0.7]
		AlterG	P < 0.001 [0.9]	HC vs. TGT	ns
		TGT	ns	AlterG vs. TGT	P = 0.003 [0.7]
unaff G 50–70% GCD	P < 0.001 [0.9]	HC	ns	HC vs. AlterG	P = 0.001 [0.9]
		AlterG	P < 0.001 [0.9]	HC vs. TGT	ns
		TGT	ns	AlterG vs. TGT	P = 0.006 [0.5]
aff RF 50–70% GCD	P < 0.001 [0.9]	HC	ns	HC vs. AlterG	P = 0.005 [0.5]
		AlterG	P < 0.001 [0.9]	HC vs. TGT	ns
		TGT	ns	AlterG vs. TGT	P = 0.002 [0.8]
unaff RF 50–70% GCD	P < 0.001 [0.9]	HC	ns	HC vs. AlterG	P < 0.001 [0.9]
		AlterG	P < 0.001 [0.9]	HC vs. TGT	ns
		TGT	ns	AlterG vs. TGT	P < 0.001 [0.9]
unaff TA 70–100% GCD	P < 0.001 [0.9]	HC	ns	HC vs. AlterG	P = 0.003 [0.7]
		AlterG	P < 0.001 [0.9]	HC vs. TGT	ns
		TGT	ns	AlterG vs. TGT	P = 0.007 [0.5]
aff G overall GCD	P < 0.001 [0.9]	HC	P < 0.001 [0.9]	HC vs. AlterG	P < 0.001 [0.9]
		AlterG	P < 0.001 [0.9]	HC vs. TGT	P < 0.001 [0.9]
		TGT	P = 0.008 [0.5]	AlterG vs. TGT	P < 0.001 [0.9]
unaff G overall GCD	P < 0.001 [0.9]	HC	P = 0.002[0.9]	HC vs. AlterG	P = 0.003 [0.9]
		AlterG	P < 0.001 [0.9]	HC vs. TGT	P = 0.004 [0.9]
		TGT	P = 0.004[0.9]	AlterG vs. TGT	P = 0.002 [0.9]
aff RF overall GCD	P < 0.001 [0.9]	HC	P = 0.004[0.9]	HC vs. AlterG	P = 0.004 [0.9]
		AlterG	P = 0.004[0.9]	HC vs. TGT	P = 0.003 [0.9]
		TGT	P < 0.001 [0.9]	AlterG vs. TGT	P = 0.001 [0.9]
unaff RF overall GCD	P < 0.001 [0.9]	HC	P = 0.001[0.9]	HC vs. AlterG	P = 0.005 [0.9]
		AlterG	P = 0.004[0.9]	HC vs. TGT	P = 0.003 [0.9]
		TGT	P = 0.003[0.9]	AlterG vs. TGT	P < 0.001 [0.9]
unaff TA overall GCD	P < 0.001 [0.9]	HC	P = 0.005[0.9]	HC vs. AlterG	P = 0.001 [0.9]
		AlterG	P = 0.002[0.9]	HC vs. TGT	P = 0.004 [0.9]
		TGT	P = 0.004[0.9]	AlterG vs. TGT	P = 0.002 [0.9]

Legend: gastrocnemius, G, rectus femoris, RF, biceps femoris, BF, tibialis anterior, TA, of affected (aff) and unaffected (unaff) lower limbs; treadmill gait training, TGT; healthy controls, HC; [E] effect size; GCD gait cycle duration.

Notably, it was observed that foot motion quickly recovered the shape and the step reproducibility (that characterizes normal gait) at the end of each AlterG session in the HC, whereas this was not the case of the patients who were provided with AlterG training.

Last, there were no significant effects of clinical-demographic characteristics on gait outcomes.

## Discussion

Both AlterG gait training and TGT provided patients with an FAC improvement of at least one point. However, as the main finding of the present study, AlterG gait training was superior to TGT in modifying the temporal variables of gait and specific muscular activation patterns. In fact, AlterG yielded a greater TS increase, cadence increase, step time decrease in the affected limb, step time increase in the unaffected limb, SSR increase in the affected limb, SSR decrease in the unaffected limb, and GQI increase (i.e., a smaller overall deviation from the average gait of a control population). Moreover, AlterG in patients targeted equally the temporal variables of the gait of both the lower limbs, whereas TGT offered more effects on the affected than the unaffected lower limb. Furthermore, AlterG induced muscle-specific (both G, both RF, and unaffected TA) and gait cycle specific (mid- and late-stance) nonlinear scaling of muscle activity as compared to TGT, with particular regard to antigravity muscles. TGT instead improved only overall muscle activation. Concerning HC, AlterG barely modified the gait cycle features and had effects on muscle activity that were limited to the overall muscle activation.

Hence, even though the patients who practiced AlterG walked as independently as the patients provided with TGT, the former treatment provided the patients walking faster, with a kinematic walking pattern closer to normal overground walking, and with more symmetric temporal variables of gait as compared to the latter treatment. These goals are not of negligible importance, as it is crucial in gait rehabilitation to provide the patient with both independent and performing gait [49].

As far as we know, this is the first study investigating the effects of LBPPSS training on temporal variables of gait in people with chronic stroke. Therefore, we can discuss our findings in comparison with those coming from other BWSTs and TGT. It has been reported that there are no significant differences between BWSTT and TGT in the patients with chronic phase of stroke with at least moderate initial ambulatory status (as in our study) [6], [50]. However, LBPPSS differs from the other BWSTs in at least two aspects: (1) the distribution of suspension forces on the body; and (2) the action of the suspension forces on both standing and swinging limb [51]. The first aspect depends on the device itself. Indeed, the other BWS devices employed to suspend patient’s weight during walking rehabilitation (including water immersion, parallel bars and walker, hand-held waist belts, and overhead suspension harness) are not characterized by the same correlation between the suspension force and the waist cross-sectional area, which accounts for the overall lifting force, and employ a from-above lifting force. The second aspect is suggested by the gathering of muscle activity changes in the mid- and late-stance phases of the gait cycle, as pointed out by our EMG data, whereas the other suspension devices seems to not allow for this activity [51]. This finding is suggestive of a correlation between AlterG-induced symmetric improvement of temporal variables of gait and the specific, more symmetric, support to the stance phase and swing initiation by part of LBPP. In particular, AlterG shaped RF and G muscles, which are central to opposing to gravity force [52]. Having bilaterally rebalanced the activation of G may have been important in gait improvement given that G acts as either a propulsive muscle during walking (by providing hip extension during the stance phase) or a muscle that prevents the foot from hitting the ground (by creating a flexion of the knee during the swing phase) [51]. Lastly, AlterG targeted unaffected TA. This is at first glance unusual, given that TA should remain relatively unaffected by the from-below, vertical force created by the LBPPSS [51]. Thus, it is likely that targeting unaffected TA resulted in a compensatory effect to establish a more stable gait dynamic, i.e., avoiding a rapid plantar flexion of the ankle during the initial stance to ensure that the forefoot clears the ground during the swing phase and positioning the ankle joint for initial ground contact. These effects also contributed to favor a more symmetric gait [53].

Further, the specific effects on muscle activation by AlterG in patients may be due to the progressive increase in gait velocity at lower biomechanical demand and higher dimensionless speeds [54], [55], [56], [57]. About that, it has been documented that increasing the speed of running while tuning the degree of LBPP seems to augment the overall locomotor muscle activity [58]. Lastly, AlterG allows patients to vary bodily posture during gait, which may have influenced lower limb muscle activation [56], [57].

Altogether, these issues may lead to a more physiologic gait pattern as compared to other non-harness BWS system (e.g., hydrotherapy) [51], [59]. Since the upward resultant air pressure force acts at or near the body’s center of mass, walking in the device will result in a more normal gait but with proportionally reduced musculoskeletal forces [51], [59].

HC were nearly insensitive to the training as compared to patients. This may depend on the more unstable spatiotemporal structure of locomotion in stroke survivors, owing to the increase of compensatory oscillating circuits driving the muscles to produce locomotion [56], [57]. Thus, weight-relief may have allowed a more efficient reshape of rhythmogenesis at the level of spinal central pattern generators receiving a deteriorated corticospinal drive [17], [60], [61], [62], [63], [64], [65] and, even, at the central level [66].

### Limitations

Other factors may come into play when dealing with LBPP, thus limiting the large-scale applicability of LBPP training. These include task-dependent features, individual compensatory strategies, and plasticity of gait-related brain and spinal networks. In addition, different levels of weight relief were not compared, selecting the most suitable level of BWS for the patient instead. Further, it is still unclear whether the effect of a therapist’s supervision may mitigate (or remove completely) an incorrect performance of the training, which obviously represents a confounding factor. Hence, further studies are needed to clarify the role of LBPP in gait training. Last, it will be necessary to ascertain whether AlterG aftereffects are long lasting with an adequate follow-up period.

## Conclusions

The application of LBPP to treadmill-based gait training seems promising in post-stroke rehabilitation. In fact, LBPPSS resulted in walking faster, large changes in the temporal walking kinematics, an improvement in functional ambulation, and a better muscle activation pattern, with particular regard to antigravity muscles as compared to TGT. However, LBPPSS has complex effects on neuromuscular activation, with non-proportional changes in body weight and muscle activity. Thus, other studies are necessary to confirm our promising findings. The knowledge of the exact gait pattern during BWSTT will be central to plan patient-tailored locomotor training. For example, the correlation between RF, G, and TA forces and gait features allows for achieving more precisely gait kinematics and kinetics during rehabilitation.

## Ethical approval

All procedures performed in studies involving human participants were in accordance with the ethical standards of the institutional and/or national research committee and with the 1964 Helsinki declaration and its later amendments or comparable ethical standards. The local Ethic Committee approved the study.

## Funding

No funding to report.

## Informed consent

Patient provided his written informed consent to study participation and publication.

## Declaration of Competing Interest

None of the authors has conflict of interest.
